# Four-parameter analysis in modified Rotarod test for detecting minor motor deficits in mice

**DOI:** 10.1186/s12915-023-01679-y

**Published:** 2023-08-17

**Authors:** Hui-Min Shan, Michael A. Maurer, Martin E. Schwab

**Affiliations:** 1 Institute for Regenerative Medicine, University of Zurich, Zurich, Switzerland; 2 Neuroscience Center Zurich, University of Zurich and ETH Zurich, Zurich, Switzerland

**Keywords:** Locomotion, Rotarod test, Traumatic brain injury, Neurological disorders

## Abstract

**Background:**

The Rotarod test with commercial apparatus is widely used to assess locomotor performance, balance and motor learning as well as the deficits resulting from diverse neurological disorders in laboratory rodents due to its simplicity and objectivity. Traditionally, the test ends when rodents drop from the accelerating, turning rod, and the only parameter used commonly is “latency to fall”. The values of individual animals can often vary greatly.

**Results:**

In the present study, we established a procedure for mice with 4 consecutive days of training with 4 trials per day and modified the testing procedure by placing the mice back on the rod repeatedly after each fall until the trial ends (5 min). Data from the fourth training day as baseline results showed that the second, third and fourth trial were more consistent than the first, probably due to habituation or learning. There was no difference between the second, third and fourth trial, two trials may be sufficient in testing. We also introduced 3 additional read-outs: Longest duration on the rod (s), Maximal distance covered (cm), and Number of falls to better evaluate the motor capacity over the 5 min of testing. We then used this 4-parameter analysis to capture the motor deficits of mice with mild to moderate traumatic brain injuries (by a weight dropping on the skull (Marmarou model)). We found that normalization of data to individual baseline performance was needed to reduce individual differences, and 4 trials were more sensitive than two to show motor deficits. The parameter of Maximal distance was the best in detecting statistically significant long-term motor deficits.

**Conclusions:**

These results show that by making adjustments to the protocol and employing a more refined analysis, it is possible to expand a widely used routine behavioral test with additional accessible parameters that detect relevant deficits in a model of mild to moderate traumatic brain injury. The modified Rotarod test maybe a valuable tool for better preclinical evaluations of drugs and therapies.

**Supplementary Information:**

The online version contains supplementary material available at 10.1186/s12915-023-01679-y.

## Background

Rotarod is a widely used test to assess locomotion and balance and the corresponding impairments and deficits associated with a variety of neurological diseases. Simple, semi-automated testing systems are available for rodents [1–4]. However, inter-laboratory reproducibility can only be achieved if the genotype, age and gender of rodents, the intensity of training and the specific parameters of the Rotarod apparatus, e.g. diameter and height of the rod, width of the lanes, composition of surface material, are comparable [5, 6]. Rotarod tests can be divided into set-speed tests [7] and accelerating Rotarod tests [8]. An important observation and drawback of the most frequently used read-out ‘first latency to fall’ is the high variability among animals [9]. In particular when studying disease models with only mild motor impairments like mild to moderate traumatic brain injury (TBI), a Rotarod testing procedure with high reproducibility and sensitivity is urgently needed.

## Results

### Four-parameter analysis of locomotor behavior of intact adult mice in the modified Rotarod test

**Fig. 1:**
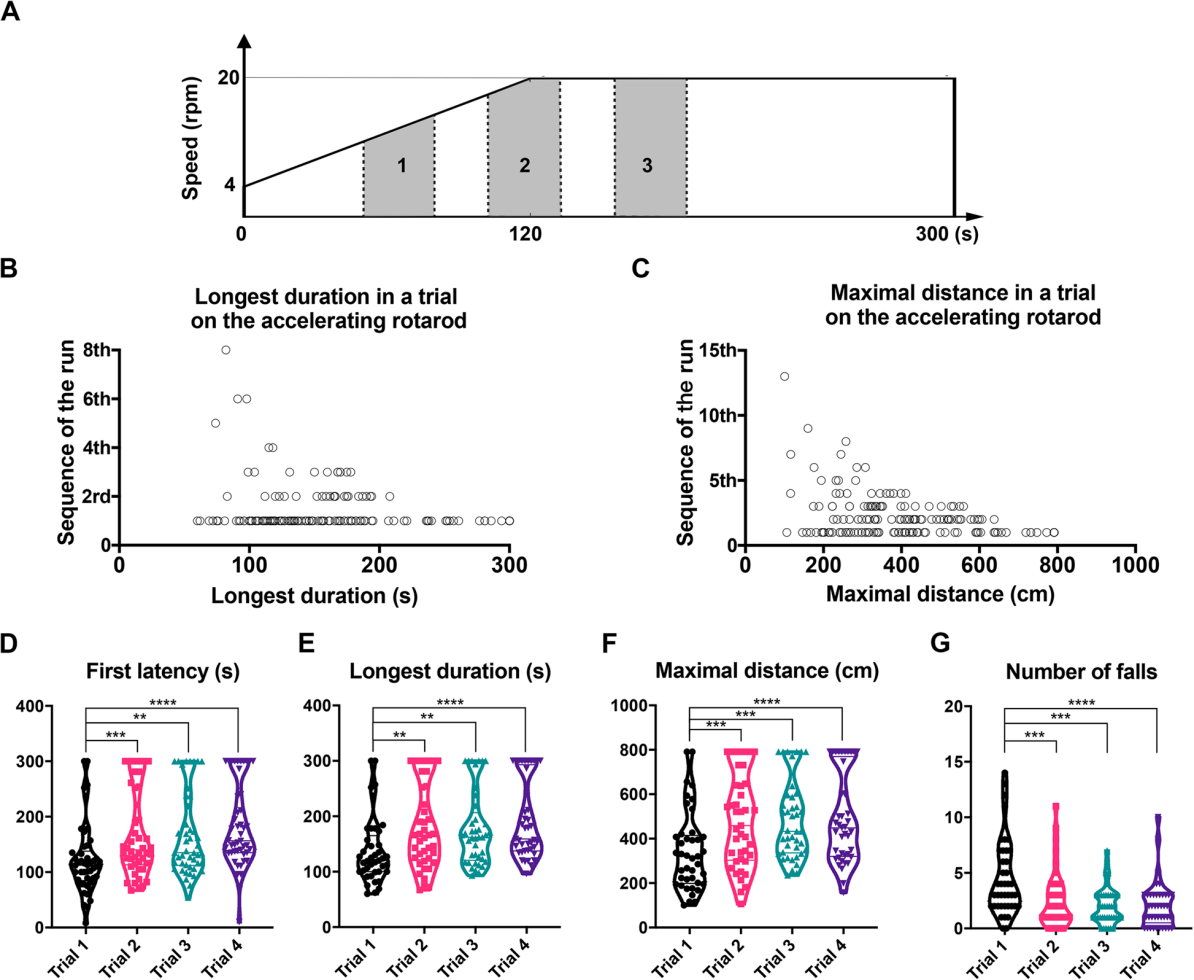
4-parameter analysis of modified Rotarod test of 48 mice on the fourth day of training. 4 trials per mouse and in total 192 trials were involved (7 trials went through testing but without recording due to technical problems). **A** Scheme illustrating time (s) on the x-axis and speed (rpm) on the y-axis in the accelerated Rotarod test. The test started with an acceleration phase from 4 to 20 rpm within the first 120 s, followed by constant speed for the subsequent 3 min. Maximal distance achieved at a given time is dependent on the rotation speed, as demonstrated by the polygons. Although three polygons with dashed lines and grey background had the same duration, polygon 3 exhibited the greatest distance due to higher speed. **B** The sequence of the run with longest duration during 5 min trial. Each run ends with a fall, each trial lasts 5 min. Ideally, mice exhibiting excellent performance, without any falls during the entire 5 min test have only one run, and in these cases, the run with the longest duration was considered as 1^st^ run. However, longest duration in 42 out of 185 trials was not in the first run. **C** The sequence of the run with maximal distance within each 5 min trial. Maximal distance in 93 out of 185 trials is not in the first run. **D**-**G** 4-parameter analysis of individual trials (Trial 1 to 4): First Latency, Longest duration, Maximal distance and Number of falls. The statistical evaluation was carried out by the Friedman test (matched data, non-parametric) followed by Dunn’s correction. *n* = 48 animals, asterisks indicate significances: **p < 0.01, ***p < 0.001, ****p < 0.0001

A modified accelerating Rotarod test protocol with a 4-parameter analysis was developed in our study. Intact adult C57BL/6J mice (*n* = 48) were trained over 4 consecutive days and tested on the fourth day. The training and testing consisted of 4 trials daily with several runs and replacement on the rod after each fall over 5 min per trial. A run ends with a fall. Ideally, mice were able to continuously walk on the rod for 5 min, but repeated falls were common. The maximum rotation speed was 12 rpm (acceleration duration: 60 s, from 4 to 12 rpm) for the first 2 days and 20 rpm (acceleration duration: 120 s, from 4 to 20 rpm) for the last 2 days (Fig. 1A). Figure 1B and C show that in 22.7% of the mice the longest duration in the 5 min testing period was not the first run in the series, and in 50.3% of the mice the run with the maximal distance in the 5 min testing period occurred not in the first but in later runs. These data showed that the mice may have a better motor capacity seen in the later runs which could be disregarded by the parameter “First latency” but captured by the parameters “Longest duration” and “Maximal distance”. The read-outs from 4 trials on the fourth training day were variable among the animals in all the trials: 4 different parameters in 4 trials all showed a non-normal distribution (detected by the Shapiro-Wilk test) with extreme values indicating the large inter-individual differences of the mice (Fig. 1D-G). When comparing the different trials, we found there were no differences between Trial 2, Trial 3 and Trial 4 in all 4 parameters, while First latency, Longest duration and Maximal distance from Trial 1 were significantly lower than in the other trials and Number of falls from Trial 1 was significantly higher than in other trials. The analysis of the first derivative across four trials revealed that the median of the first derivative from the inter-trial 1.5 (Trial 1 to Trial 2) was the highest compared to other inter-trials in First Latency, Longest duration and Maximal distance, and there were significant differences in the parameter of “Number of falls” from the inter-trial 1.5 compared to other inter-trials (*p* < 0.01). No differences were found between inter-trial 2.5 and inter-trial 3.5. These results suggest an improved motor coordination starting from Trial 2 and consistent data in Rotarod test can be gained from Trial 2 to Trial 4 (Additional file 1: Fig. S1A-D).

### No age and gender differences in the modified Rotarod test by 4-parameter analysis

**Fig. 2:**
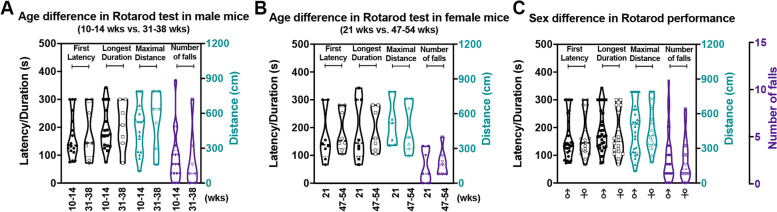
No age and gender differences in the modified Rotarod test analyzed by 4 parameters. **A** 4-parameter read-outs in Trial 2 at baseline testing in young adult males (10–14 wks, *n* = 19, solid symbols) vs. middle-aged males (31–38 wks, *n* = 7, hollow symbols). **B** 4-parameter read-outs in young-adult female mice (21 wks, *n* = 7, solid symbols) vs. middle-aged (47–54 wks, *n* = 7, hollow symbols) females. **C** 4-parameter read-outs for adult males (*n* = 28, solid symbols) vs. females (*n* = 18, hollow symbols). First latency and Longest duration are shown in seconds (s) on the left Y axis, Maximal distance is shown in centimeters (cm) on the first right Y axis and Number of falls is shown in on the last Y axis. Statistical evaluations were done by the Kruskal-Wallis test followed by Dunn’s correction for non-matched and non-parametric data. The *p*-values obtained from the analysis were greater than 0.99, indicating no significant difference among the groups

To investigate a possible effect of age on motor performance in the modified Rotarod test, young adult and middle-aged male and female C57BL/6J mice were tested. As shown in Fig. 2A and B, no significant age-related differences were found, neither for males (10–14 (weeks) wks vs. 31–38 wks) nor for females (21 wks vs. 47–54 wks). When comparing young adult and middle-aged males, the following data points were observed (presented as median with interquartile range (IQR)): First latency in Trial 2 to Trial 4 was 134.0 (128.0) vs. 143.0 (218.0) s, Longest duration was 170.0 (108.0) vs. 208.0 (158.0) s, Maximal distance was 527.5 (363.7) vs. 638.0 (493.0) cm, and Number of falls was 2.0 (2.5) vs. 1.0 (4.0) (Fig. 2A). Also, no significant differences were observed between young and middle-aged females. The readouts for First latency were 134.0 (213.0) s in 21-week-old females vs. 161.0 (162) s in 47–54 weeks-old females, for Longest duration was 134.0 (213.0) vs. 161.0 (162.0) s, for Maximal distance were 521.2 (455.3) vs. 398.9 (433.3) cm, and for Number of falls 1.0 (4.0) vs. 2.0 (4.0) (Fig. 2B).

Furthermore, we examined a potential impact of gender on the locomotor performance by comparison of male and female mice (Fig. 2C). The differences between males and females were not found to be significant by the Kruskal-Wallis test: First latency 135.5 (123.5) s in males vs. 149.0 (140.0) s in females; Longest duration 168.0 (103.5) vs. 149.0 (140.0) s; Maximal distance 505.6 (370.8) vs. 410.0 (310.9) cm; Number of falls 2.0 (3.0) vs. 1.5 (3.0).

### 4-parameter analysis detects long-term motor deficits in mice after mild to moderate traumatic brain injury

**Fig. 3:**
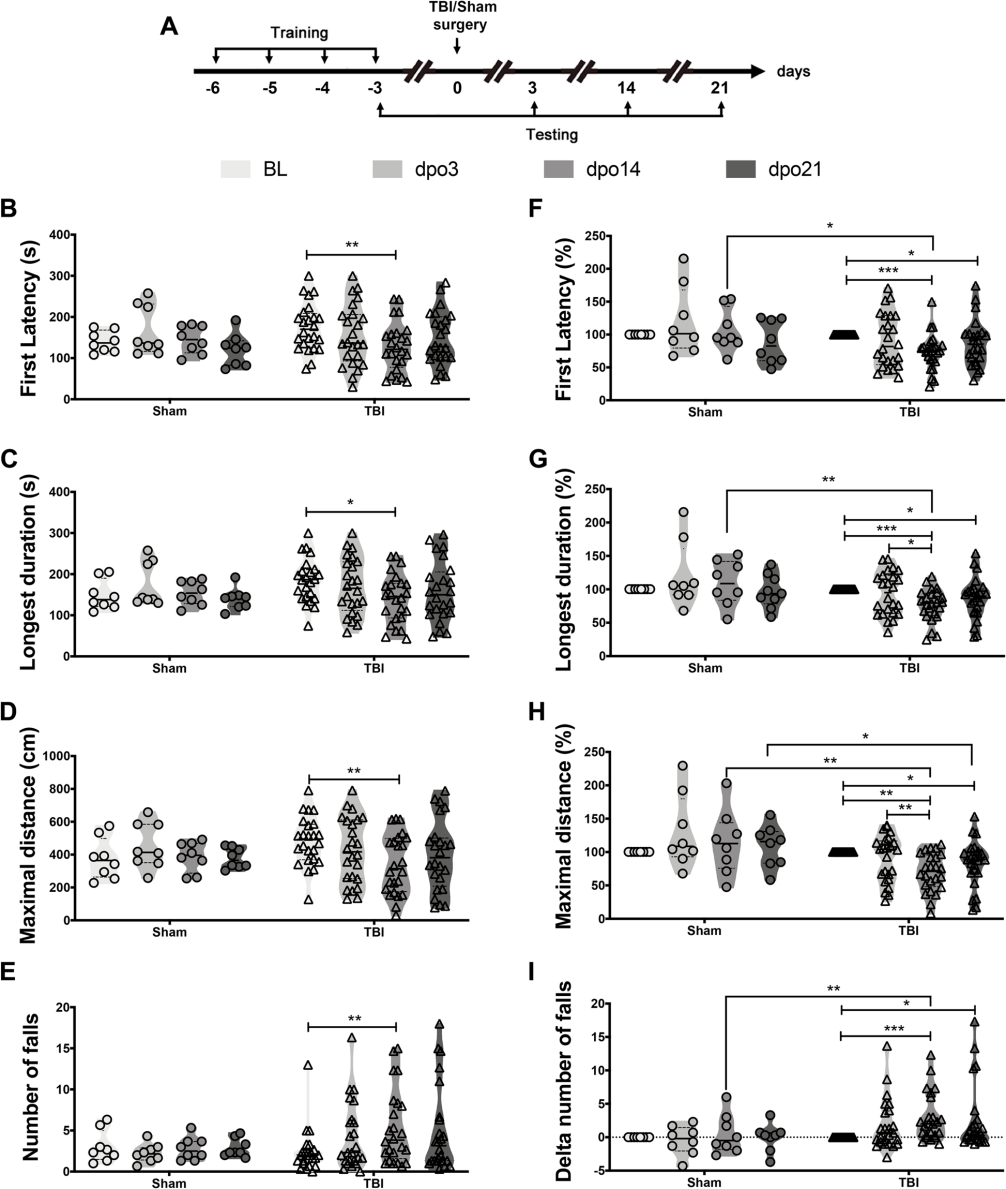
4-parameter analysis in detecting minor motor deficits in comparison of Sham and TBI mice. **A** Study timeline with the Rotarod test in the TBI and Sham group: BL testing is performed 3 days prior to the operation and others at dpo3, dpo14, dpo21. **B**-**E** Raw averaged data from Trial 2 to Trial 4 at different time points: First latency to fall (s), Longest duration on rod (s), Maximal distance on rod (cm) and Number of falls. **F-H** Normalized data with normalization to the average of Trial 2 to Trial 4 in baseline testing individually: First latency to fall (%), Longest duration (%) and Maximal distance on rod (%). I) Normalized data with normalization by subtracting to the average of Trial 2 to Trial 4 in baseline testing individually: delta number of falls. Statistical analysis was carried out by the Kruskal-Wallis test followed by Dunn’s correction. *n* = 8 in Sham group with circular symbols and *n* = 24 in TBI group with triangle symbols, asterisks indicate significances: *p < 0.05, **p < 0.01, ***p < 0.001

We applied our newly modified Rotarod test with a 4-parameter analysis for the comparison of TBI and Sham mice (anesthesia, helmet, but no impact). Training with a maximum of 12 rpm was done on day -6 and -5 before TBI or Sham operation, followed by training at a maximum of 20 rpm at days -4 and -3. The training on the last day (day -3) was recorded as the Baseline (BL) of individual mice. We then performed longitudinal testing at 3, 14 and 21 days post operation (dpo) (Fig. 3A). As shown in Fig. 3B-E, raw values varied over a large range in all the parameters and conditions. To reduce individual differences, we normalized First latency to fall, Longest duration and Maximal distance on rod by dividing the post-operation performance by the respective individual baseline results (Fig. 3F-H). Number of falls was normalized by subtracting the post-operation performance from the baseline as “Delta number of falls” (Fig. 3I). To calculate the difference between the Sham and TBI groups, we first performed a comparison of individual trials (Additional file 2: Table S1). We found that averaging the values of Trial 2, 3 and 4 decreased the power of extreme values between trials for individual mice, making the parameters First latency, Longest duration and Maximal distance normally distributed. Averaged values of Trials 2 to 4 were the most sensitive readouts to detect motor deficits within the TBI group and between the TBI and Sham groups.

The averaged data of the Trial 2 to 4 were shown in Fig. 3. Sham animals did not show significant differences between baseline and post operation testing (dpo3, dpo14 and dpo21). In contrast, significant differences were seen in the TBI animals after injury, interestingly with a protracted time course where the maximal deficits were found between dpo14 and dpo21 (Fig. 3). The deficits were visible in the comparison with the TBI animal baseline values as well as in the comparison with the Sham animals. These results showed that motor deficits can be found in the TBI mice at later time points after the injury. The parameters Longest duration, Maximal distance and Number of falls can better capture these deficits than First Latency to fall only. Maximal distance showed the higher sensitivity among all the parameters.

## Discussion

The Rotarod test is widely used in testing the effects of drugs and different types of brain damage but is notorious for high variability of the data obtained. Ways to reduce variability leading to more reliable and consistent read-outs are therefore of high interest. In our study, we modified the test by placing mice back on the rod after a fall repeatedly to evaluate the rodents’ locomotor capacity over a trial period of 5 min. We ran 4 training or testing trials per day. We also added 3 new parameters to the original parameter “First Latency to fall”: “Longest duration” (time of the longest run per trial), “Maximal distance” (Maximum distance covered in a given run per trial) and Number of falls per 5-min trial. Only Number of falls has been used before in a few studies [10, 11]. With the incorporation of additional parameters, a better description of the motor behavior on the accelerating Rotarod was possible, including also aspects of strength, fatigue and motor learning. This approach offers a more comprehensive evaluation compared to relying solely on First Latency to fall as an indicator of motor impairment. Normalization of readouts to the individual baseline data and averaging of trials are useful additional ways to reduce variability and obtain a solid reflection of the animals’ motor capabilities.

### Highly interdependent but not redundant parameters in Rotarod running instead of a single run/first latency to fall

We assessed the four chosen parameters in each run over the 5 min testing period in intact adult mice. We found that maximal performances occurred in runs which were not the first one in many instances. The intrinsic nature of these parameters makes them dependent on each other (determined by Spearman correlation, *p* < 0.0001, data not shown). Specifically, mice with higher values of “First Latency” are more likely to have higher values of “Longest duration” and “Maximal distance”, while having lower values of “Number of falls”, indicating better performance. However, it is important to note that these parameters are not redundant. Conceptually, “Longest duration” represents the motor endurance and muscle fatigue of mice on the rod over the entire 5-min period, taking into account later runs, which is disregarded by “First Latency”. Similarly, “Maximal distance” considers speed (accelerating at the beginning and constant in the end) with the reflection of muscle kinetics to compute motor capacity over the 5-min period [12]. “Number of falls” may incorporate motor learning processes of strength and coordination. In practical terms, when comparing TBI and Sham groups, the parameter “Maximal distance” proved to be the most effective in detecting statistically significant long-term motor deficits. This is likely because “Maximal distance” takes into account both later runs and the speed of the runs over the entire test period. Therefore, a 5 min training/testing period with repeated runs after each fall with 4-parameter analysis gave a more reliable picture than just the first run ever.

### Repeated 5 min trials per training/testing day

We compared the behavior of the mice as reflected by the four parameters "First latency to fall", "Longest duration of a run", "Maximal distance of a run" and "Number of falls" in Trial 1 to 4 after four days of training and found that trial 1 had the greatest variability in the data, whereas the outcomes of trials 2 to 4 were very similar and consistent. Thus, exclusion of Trial 1 for the final assessment of motor performance is meaningful in intact mice, Trial 2 reflected the full capacity of Rotarod performance. In the TBI animals, however, the trial to trial comparison showed that four trials can give a more precise picture with clearer differences among the conditions than the analysis of Trial 1 or 2 only.

### Age and gender differences

No significant differences could be observed in our data between young adult (10–21 wks) and middle-aged mice (31–54 wks). Decreased Rotarod performance with age has been reported before in some rodent models, but mostly with animals of more advanced age [13–15]. In adult male and female mice, no significant gender differences were detected in wild-type mice in our study. Previous studies on the Rotarod test have shown variable findings regarding gender differences. While some studies align with our results [16, 17], other studies indicated that female mice of the same age tend to outperform males, likely due to their lighter weights [5, 18, 19]. It is important to note that the comparison between male and female mice is confounded by variability introduced by differences in age and the associated changes in weight. This confounding factor may also represent a limitation in interpreting and generalizing our finding of no significant gender differences in this study.

### Deficits in Rotarod performance after closed head TBI

TBI is a major medical problem worldwide, and most TBIs are of the closed head injury type [20–22]. TBI can lead to permanent impairments of cognitive, memory, motor learning, and psychosocial functions [23]. Motor dysfunction is of obvious high importance as it can influence performance in many other tests, e.g., for cognitive, anxiety or social behaviors and up to 30% of mild traumatic brain injury patients show minor deficits in fine movement, balance and coordination [24, 25]. Mild or moderate TBI in mice was reported to not affect the performance on the accelerating Rotarod [26, 27]. This contrasts with the present results where we found clear impairments in several of the observed parameters after a mild to moderate weight drop injury. Differences in the lesion severity and the more sensitive evaluation of the mouse behavior in the Rotarod test applied here may account for this discrepancy. The present results suggest that locomotor impairments are a relevant consequence of closed head TBI also in mice.

### Delayed manifestation of impaired locomotor performance after TBI

After acute traumatic spinal cord injury or after stroke, motor impairments manifest themselves immediately; maximal deficits are often observed in the first few days after the injury [28, 29]. This is in contrast to our present observations in the mild to moderate TBI mice, where the Rotarod behavior was often only mildly or not affected at 3 days after the impact, but increased over time and became maximal at 14 to 21 days after injury. This is in line with several recent publications which showed the delayed and progressed appearance of different behavioral deficits in TBI, paralleled by diffuse, widespread blood brain barrier dysfunction and inflammatory processes like microglia activation as well as synapse loss throughout the brain [30–33].

### Limitations of this study

1) Applicability: While the modified procedures and analysis could be applied to various Rotarod tests, regardless of genotype, training intensity, and different parameters of the Rotarod apparatus from different companies, it is important to acknowledge its specific applicability. The modified protocol has shown responsiveness in milder forms of traumatic brain injury, and it may be useful to assess motor deficits of mild to moderate type in other neurological disease models. However, in animal models with severe motor deficits, such as large strokes, the primary measure of First latency to fall may be adequate and sufficient. 2) Data analysis: For the purpose of conducting a straightforward and efficient comparison between post-injury results at a specific time point and baseline results, an alternative method such as the one-sample t-test (parametric) or the sign test (non-parametric) can be employed. This is particularly applicable when conducting comparisons after normalization, considering that there is no variance at the baseline (with a delta number of falls of 0 and other parameters at 100%). In addition, the potential exploration of combining the four parameters and addressing dependent redundancy through principal component analysis could offer valuable insights in terms of simplifying data interpretation and enhancing the understanding of complex relationships among the parameters. 3) Disparity in sample size between Sham and TBI: The differences in sample sizes in this study was due to factors like mouse breeding and limitations imposed by the Animal License based on the principles of the 3Rs (Replacement, Reduction, and Refinement). We conducted a post-hoc power analysis to ensure that our sample size was adequate and had a reasonable likelihood of yielding statistically significant results. Additionally, we employed the Kruskal–Wallis test as an appropriate statistical test, given its ability to mitigate the impact of varying sample sizes between the groups by using ranks instead of raw data values. This approach helped to account for the unequal sample sizes and maintain the integrity of our statistical analysis.

## Conclusions

The modifications of the accelerating Rotarod test introduced in this study allow to assess a broader range of physiological aspects of locomotion as well as minor motor deficits caused. e.g. by mild to moderate injuries. As shown by the analysis of closed head TBI mice, they allow to reliably observe locomotor impairments which were missed in earlier studies when using only the traditional "First latency to fall" parameter. These simple modifications can be broadly applied to all available Rotarod tests.

## Methods

### Animals and reagents

A total of 58 adult C57/BL6J (25–35 g, 3–12 months old, 21 females and 37 males, Charles River, Germany) were used in this project, 10 of 58 were excluded because of bad performance in Rotarod baseline testing in spite of sufficient training. The exclusion criteria were Number of falls ≥ 10 in 2 consecutive trials at baseline testing. Animals were housed in groups of 3 to 5 in open ventilated cages with free access to food and water on a 12-h dark/light cycle. This study complied with the ARRIVE guidelines, and all experimental procedures were approved by the local veterinary office, Canton of Zurich, Switzerland. 1% (wt/vol) Virkon S (Arovet, Switzerland) in water was used for the disinfection of hands, surfaces and instruments, 75% (vol/vol) ethanol (Reusschemie, Switzerland) in water to clean the apparatus after each trial and kitchen paper to remove excreta (feces) from the rodents after ethanol spraying.

### TBI surgery

We used Marmarou’s weight-drop model for diffuse axonal injury TBI as this method is suitable for studying brain trauma arising from falls or sports or traffic accidents [34]. Briefly, TBI was induced by dropping a 90–120 g metal rod through a plastic tube from 45–70 cm height onto a ‘helmet’ (metal plate, diameter = 12 mm, the center of the helmet is Anterior-Posterior (AP) with regard to bregma: + 2.2 mm; Medial-Lateral (ML): 0 mm glued to the scull of the mice [35, 36]. The mice were placed on a sponge at 12-degree inclination to ensure that the skull of the mice was parallel to the ground, so that the impact of the weight as well as the rebound occurred perpendicularly to the center of the helmet. Mice were anesthetized with fentanyl–midazolam–medetomidine anesthesia (Fentanyl, 0.05 mg/kg, Sintetica; Midazolam, 5 mg/kg, Sintetica; Medetomidine, 0.5 mg/kg, Grion Pharma) intraperitoneally before surgery [37]. Local anesthetic was applied 3 min prior to the skin incision for the helmet placement using a subcutaneous injection of lidocaine (10 mg/kg, Streuli Pharma). To revert the anesthesia when the impact was complete (at about 20 min.), Atipamezole (2.5 mg/kg, Grion pharma) and Flumazenil (0.5 mg/kg, CPS Cito Pharma) were injected subcutaneously. As analgesic, all animals received Carprofen (5 mg/kg body weight, Zoetis) subcutaneously every 12 h for a total of 3 days after surgery. The Sham group went through the same operation process, including anesthesia, skin incision, postoperative care, but without the weight drop injury. Following the last behavioral testing, the animals were euthanized through intraperitoneal administration of pentobarbital (600 mg/kg body weight, Streuli Pharma).

### Equipment

Rotarod apparatus (TSE systems) with an automatic timer. The height of the turning rod from the base plate was 29 cm with a 5 cm soft sponge on the base plate. The width of the individual compartments on the rod was 9 cm which ensured that mice had enough space to move freely without turning. The diameter of the rod was 3 cm with a rippled surface. The speed of the rod was accelerated from 4 to 12 rpm (training days 1, 2) or from 4 to 20 rpm (days 3 and 4) for training and testing.

### Procedures

1. **Handling in the behavioral room** (Timing: 10 min/day): 2 weeks’ handling with 10 min per day before the rodents go into experiments. Handling starts with placing hands on the cage, interacting with mice by fingers, cupping mice on the open hands and finally using transparent tunnels to reduce anxiety and aversion towards the experimenters, as well as the anxiety stemming from the novelty of the behavioral room.
2. **Habituation in the behavioral room** (Timing: 5 min): Adjust the room brightness before bringing the mice from their housing room to the behavioral room 5 min before training or testing. Lights that are too light could cause anxiety for mice. In contrast, it is difficult for experimenters to perform and record results in low light. In our test, we use an intermediate brightness around 600 lx. Each lane of the Rotarod apparatus should be similarly bright.
3. **Habituation to the Rotarod apparatus** (Timing: 2 min): Pick up the mice by cupping hands under them and let the mice sit on the hands. Gently place the mice on the rod with 4 limbs touching the rod facing in the direction of the rotating rod, with its back to the experimenter. On the first training day, rodents will explore freely and stand on the stationary rod for 2 min before the first trial.
4. **Training days 1 and 2** (Timing: 1 h): Training up to 12 rpm: accelerate from 4 to 12 rpm during the first 1 min, then keep constant for 4 min. Gently pick mice up and replace them on the rod if they fell. 4 trials of 5 min. each per day for each animal, with pauses of 8 min between the trials. Clean and disinfect the equipment with 75% ethanol and kitchen paper before placing the next mice.
5. **Training day 3** (Timing: 1 h): Training up to 20 rpm: accelerate from 4 to 20 rpm for the first 2 min, then keep constant for 3 min, 4 trials of 5 min. each for each animal with 8 min intervals.
6. **BL testing day 4** (Timing: 1 h): As described in Training day 3. Place a timer next to the experimenter. The time for each mouse to fall from the rod is recorded for each run.
7. **Testing after injury** (around 1 h): As described in BL testing day 4.

### Real-time counting vs. counting from recorded videos

All the data presented in the main body of this article were obtained through real-time counting and time measurements. However, we also tried to count from recorded videos to investigate the difference between the two methods. The results showed that the falling time difference obtained by the two approaches was -1 to 2 s from 6 different trials of 6 mice (Additional file 3: Fig. S2A). Acquired from recorded videos, data from 30 periods showed the time difference between “Pick-Fall” having a median of 3 and interquartile of 2 (s), “Release-Fall” having a median of 5 and interquartile of 3 (s) and “Release-Pick” having a median of 1 and interquartile of 2 (s) (Additional file 3: Fig. S2B). The two highest values of 8 in “Release-Fall” happened when two mice dropped at the same time, one experimenter needed to pick them sequentially. The acceptable differences between the two methods and the much higher time and effort required by the video analyses led us to decide for manual counting for the type of study shown here.

### Data analysis

Statistical analyses were performed by using GraphPad Prism 8.2.1, Python 3.9.12 and G*Power. A hoc power analysis using G*Power tool was conducted to ensure the adequacy of our sample size and the likelihood of obtaining statistically significant results [38, 39]. All data were tested for normal distribution by using the Shapiro-Wilk test. For comparing the difference between 4 trials at BL testing, the non-parametric Friedman test with Dunn’s correction was used for pairwise comparisons (Fig. 1 and Additional file 1: Fig. S1). The Kruskal-Wallis test followed by Dunn’s correction was applied for different parameters and the comparison between trials and between different age and gender groups as well as the differences between the TBI and Sham group at the same time point or the differences between time points within the groups for all the parameters (Figs. 2 and 3 and Additional file 2: Table S1). Data are presented as median with IQR calculated by the difference between upper third quartile (Q3) and lower first quartile (Q1). Asterisks indicate significances: *p < 0.05, **p < 0.01, ***p < 0.001, ****p < 0.0001.

Distance is measured by using the formula below: Distance = Area (rpm × falling-time-seconds) × 60 × 2 π r, Area is illustrated in Fig. 1A. Radius of the rod in our apparatus is equal to 1.5 cm. 4 parameters can be automatically calculated using Python Shapely library. Python scripts were shown as below:
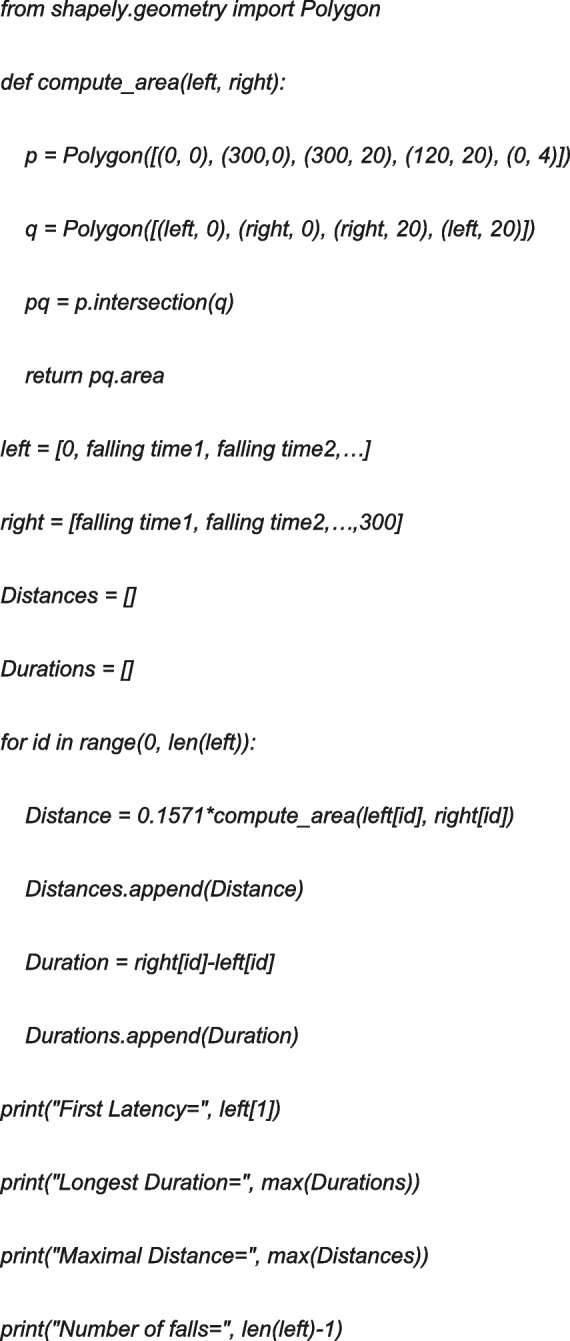


### Supplementary Information

**Additional file 1: Fig. S1.** First derivative across 4 trials in 4 parameters on the fourth day of training. The parameters assessed here were: A First Latency, B Longest duration, C Maximal distance and D Number of falls. The first derivative was calculated using the formula for the individual mice: First derivative _x+0.5_ = Trial _x+1_-Trial _x_. for the inter-trial 1.5 (Trial 1 to Trial 2), inter-trial 2.5 (Trial 2 to Trial 3) and inter-Trial 3.5 (Trial 3 to Trial 4). The black lines stretched from violin plots were defined as third quartile, median and first quartile. *n* = 48 animals, statistical analysis was performed by the Kruskal-Wallis test followed by Dunn’s correction, asterisks indicate significance level: **p < 0.01.

**Additional file 2: Table S1.** Trials comparison of Sham and TBI mice at different time points. Data at BL, dpo3, dpo14 and dpo21 are analyzed by the Kruskal-Wallis test (non-parametric) and Shapiro-Wilk Test (Normal distribution test): Trial 1 only, Trial 2 only, Trial 3 only, Trial 4 only, Averaged values in Trial 2 to 3 and Averaged Trial 2 to 4. Each Trial had 8 parameters: raw or individual normalized data in First Latency, Longest duration, Maximal distance and Number of falls. Cells filled with grey background in non-parametric test indicate significances, and the darker the grey, the more significant the difference. Asterisks indicate significances: *p < 0.05, **p < 0.01, ***p < 0.001, ****p < 0.0001. Biggest motor deficits within the TBI group showed at dpo14 compared to BL testing detected in all the trials. Starting from Trial 3, we can detect significant difference in 4 parameters not only within the TBI group, but also at the same time point between the TBI and the Sham group: Trial 3 only, Trial 4 only, Averaged Trial 2 to 3 and Averaged Trial 2 to 4. Averaged Trial 2 to 4 showed more sensitive and normal distributed readouts able to detect difference between Sham and TBI at dpo21. With raw data from 4 parameters, data are highly variable in the group (non-normally distributed), we cannot find any difference between TBI and Sham group except a slight difference at BL testing detected by Maximal distance (*p* = 0.043).

**Additional file 3: Fig. S2.** The difference between real-time counting and counting from recorded video. A The difference in the falling time by 2 different methods (Difference = falling time counted real-time - falling time counted from recorded video) from in total of 6 5-min videos (*n* = 6 animals). B The time difference between the time to pick the mice and the time of falling (Pick-Fall), the time of releasing the mice from hands to the time of falling (Release-Fall) and the time difference between Release and pick (Release-Pick) in a total of 30 periods. The black lines stretched from violin plots were defined as third quartile, median and first quartile.

## Data Availability

The authors declare that all data generated or analyzed during this study are included in this published article and its supplementary information files. The data presented in Fig. 3 and Additional file 2: Table S1 have been uploaded and shared through Figshare (http://doi.org/10.6084/m9.figshare.23899353).

## Abbreviations

AP: Anterior-Posterior
BL: Baseline
dpo: Day post operation
ML: Medial-Lateral
TBI: Traumatic brain injury
IQR: Interquartile range
Q1: Lower first quartile
Q3: Upper third quartile
wks: Weeks

## References

[1] Shan HM, Zang M, Zhang Q, Shi RB, Shi XJ, Mamtilahun M, Liu C, Luo LL, Tian X, Zhang Z (2020). Farnesoid X receptor knockout protects brain against ischemic injury through reducing neuronal apoptosis in mice. J Neuroinflammation.

[2] Shiotsuki H, Yoshimi K, Shimo Y, Funayama M, Takamatsu Y, Ikeda K, Takahashi R, Kitazawa S, Hattori N (2010). A rotarod test for evaluation of motor skill learning. J Neurosci Methods.

[3] Scholz J, Niibori Y, Frankland PW, Lerch JP (2015). Rotarod training in mice is associated with changes in brain structure observable with multimodal MRI. Neuroimage.

[4] Wagner JM, Sichler ME, Schleicher EM, Franke TN, Irwin C, Low MJ, Beindorff N, Bouter C, Bayer TA, Bouter Y (2019). Analysis of motor function in the Tg4-42 mouse model of Alzheimer’s disease. Front Behav Neurosci.

[5] Eltokhi A, Kurpiers B, Pitzer C (2021). Comprehensive characterization of motor and coordination functions in three adolescent wild-type mouse strains. Sci Rep.

[6] Mancuso R, Olivan S, Mancera P, Pasten-Zamorano A, Manzano R, Casas C, Osta R, Navarro X (2012). Effect of genetic background on onset and disease progression in the SOD1-G93A model of amyotrophic lateral sclerosis. Amyotroph Lateral Scler.

[7] Dunham NW, Miya TS (1957). A note on a simple apparatus for detecting neurological deficit in rats and mice. J Am Pharm Assoc Am Pharm Assoc.

[8] Jones BJ, Roberts DJ (1968). The quantiative measurement of motor inco-ordination in naive mice using an acelerating rotarod. J Pharm Pharmacol.

[9] Deacon RM (2013). Measuring motor coordination in mice. J Vis Exp.

[10] Tancheva LP, Lazarova MI, Alexandrova AV, Dragomanova ST, Nicoletti F, Tzvetanova ER, Hodzhev YK, Kalfin RE, Miteva SA, Mazzon E (2020). Neuroprotective mechanisms of three natural antioxidants on a rat model of Parkinson’s disease: a comparative study. Antioxidants (Basel).

[11] Han SR, Kang YH, Jeon H, Lee S, Park SJ, Song DY, Min SS, Yoo SM, Lee MS, Lee SH (2020). Differential expression of miRNAs and behavioral change in the cuprizone-induced demyelination mouse model. Int J Mol Sci.

[12] Stoquart G, Detrembleur C, Lejeune T (2008). Effect of speed on kinematic, kinetic, electromyographic and energetic reference values during treadmill walking. Neurophysiol Clin.

[13] Graber TG, Ferguson-Stegall L, Kim JH, Thompson LV (2013). C57BL/6 neuromuscular healthspan scoring system. J Gerontol A Biol Sci Med Sci.

[14] Hernandez AR, Truckenbrod LM, Campos KT, Williams SA, Burke SN (2020). Sex differences in age-related impairments vary across cognitive and physical assessments in rats. Behav Neurosci.

[15] Noda S, Sato S, Fukuda T, Tada N, Hattori N (2020). Aging-related motor function and dopaminergic neuronal loss in C57BL/6 mice. Mol Brain.

[16] Mao JH, Langley SA, Huang Y, Hang M, Bouchard KE, Celniker SE, Brown JB, Jansson JK, Karpen GH, Snijders AM (2015). Identification of genetic factors that modify motor performance and body weight using Collaborative Cross mice. Sci Rep.

[17] Munoz-Castaneda R, Diaz D, Avila-Zarza CA, Alonso JR, Weruaga E (2014). Sex-influence of nicotine and nitric oxide on motor coordination and anxiety-related neurophysiological responses. Psychopharmacology.

[18] Kovacs AD, Pearce DA (2013). Location- and sex-specific differences in weight and motor coordination in two commonly used mouse strains. Sci Rep.

[19] Tucker LB, Fu AH, McCabe JT (2016). Performance of male and female C57BL/6J mice on motor and cognitive tasks commonly used in pre-clinical traumatic brain injury research. J Neurotrauma.

[20] Maas AIR, Menon DK, Adelson PD, Andelic N, Bell MJ, Belli A, Bragge P, Brazinova A, Buki A, Chesnut RM (2017). Traumatic brain injury: integrated approaches to improve prevention, clinical care, and research. Lancet Neurol.

[21] Thomas RE, Alves J, Vaska Mlis MM, Magalhaes R (2017). Therapy and rehabilitation of mild brain injury/concussion: systematic review. Restor Neurol Neurosci.

[22] Dewan MC, Rattani A, Gupta S, Baticulon RE, Hung YC, Punchak M, Agrawal A, Adeleye AO, Shrime MG, Rubiano AM, Rosenfeld JV, Park KB. Estimating the global incidence of traumatic brain injury. J Neurosurg. 2018;130(4):1080–97. 10.3171/2017.10.JNS17352. 10.3171/2017.10.JNS17352 29701556

[23] Ding Y, Yao B, Lai Q, McAllister JP (2001). Impaired motor learning and diffuse axonal damage in motor and visual systems of the rat following traumatic brain injury. Neurol Res.

[24] Basford JR, Chou LS, Kaufman KR, Brey RH, Walker A, Malec JF, Moessner AM, Brown AW (2003). An assessment of gait and balance deficits after traumatic brain injury. Arch Phys Med Rehabil.

[25] Slobounov S, Sebastianelli W, Simon R (2002). Neurophysiological and behavioral concomitants of mild brain injury in collegiate athletes. Clin Neurophysiol.

[26] Bolte AC, Dutta AB, Hurt ME, Smirnov I, Kovacs MA, McKee CA, Ennerfelt HE, Shapiro D, Nguyen BH, Frost EL (2020). Meningeal lymphatic dysfunction exacerbates traumatic brain injury pathogenesis. Nat Commun.

[27] McAteer KM, Corrigan F, Thornton E, Turner RJ, Vink R (2016). Short and long term behavioral and pathological changes in a novel rodent model of repetitive mild traumatic brain injury. PLoS One.

[28] Lindau NT, Banninger BJ, Gullo M, Good NA, Bachmann LC, Starkey ML, Schwab ME (2014). Rewiring of the corticospinal tract in the adult rat after unilateral stroke and anti-Nogo-A therapy. Brain.

[29] Hofer AS, Scheuber MI, Sartori AM, Good N, Stalder SA, Hammer N, Fricke K, Schalbetter SM, Engmann AK, Weber RZ (2022). Stimulation of the cuneiform nucleus enables training and boosts recovery after spinal cord injury. Brain.

[30] Alawieh A, Chalhoub RM, Mallah K, Langley EF, York M, Broome H, Couch C, Adkins D, Tomlinson S (2021). Complement drives synaptic degeneration and progressive cognitive decline in the chronic phase after traumatic brain injury. J Neurosci.

[31] Krukowski K, Chou A, Feng X, Tiret B, Paladini MS, Riparip LK, Chaumeil MM, Lemere C, Rosi S (2018). Traumatic brain injury in aged mice induces chronic microglia activation, synapse loss, and complement-dependent memory deficits. Int J Mol Sci.

[32] van Vliet EA, Ndode-Ekane XE, Lehto LJ, Gorter JA, Andrade P, Aronica E, Grohn O, Pitkanen A (2020). Long-lasting blood-brain barrier dysfunction and neuroinflammation after traumatic brain injury. Neurobiol Dis.

[33] Richmond-Hacham B, Izchak H, Elbaum T, Qubty D, Bader M, Rubovitch V, Pick CG (2022). Sex-specific cognitive effects of mild traumatic brain injury to the frontal and temporal lobes. Exp Neurol.

[34] Marmarou A, Foda MA, van den Brink W, Campbell J, Kita H, Demetriadou K (1994). A new model of diffuse brain injury in rats. Part I: pathophysiology and biomechanics. J Neurosurg.

[35] Ma X, Aravind A, Pfister BJ, Chandra N, Haorah J (2019). Animal models of traumatic brain injury and assessment of injury severity. Mol Neurobiol.

[36] Kane MJ, Angoa-Perez M, Briggs DI, Viano DC, Kreipke CW, Kuhn DM (2012). A mouse model of human repetitive mild traumatic brain injury. J Neurosci Methods.

[37] Fleischmann T, Jirkof P, Henke J, Arras M, Cesarovic N (2016). Injection anaesthesia with fentanyl-midazolam-medetomidine in adult female mice: importance of antagonization and perioperative care. Lab Anim.

[38] Faul F, Erdfelder E, Buchner A, Lang AG (2009). Statistical power analyses using G*Power 3.1: tests for correlation and regression analyses. Behav Res Methods.

[39] Faul F, Erdfelder E, Lang AG, Buchner A (2007). G*Power 3: a flexible statistical power analysis program for the social, behavioral, and biomedical sciences. Behav Res Methods.

