# Supplementary material for: Four-parameter analysis in modified Rotarod test for detecting minor motor deficits in mice
Source: BMC Biol. 2023 Aug 17;21:177. doi: 10.1186/s12915-023-01679-y (PMC10433596; doi:10.1186/s12915-023-01679-y)
Supplement: Supplementary file 1 — Additional file 1: Fig. S1. First derivative across 4 trials in 4 parameters on the fourth day of training. The parameters assessed here were: A First Latency, B Longest duration, C Maximal distance and D Number of falls. The first derivative was calculated using the formula for the individual mice: First derivative x+0.5 = Trial x+1-Trial x. for the inter-trial 1.5 (Trial 1 to Trial 2), inter-trial 2.5 (Trial 2 to Trial 3) and inter-Trial 3.5 (Trial 3 to Trial 4). The black lines stretched from violin plots were defined as third quartile, median and first quartile. n = 48 animals, statistical analysis was performed by the Kruskal-Wallis test followed by Dunn’s correction, asterisks indicate significance level: **p < 0.01. [file 12915_2023_1679_MOESM1_ESM.pdf]

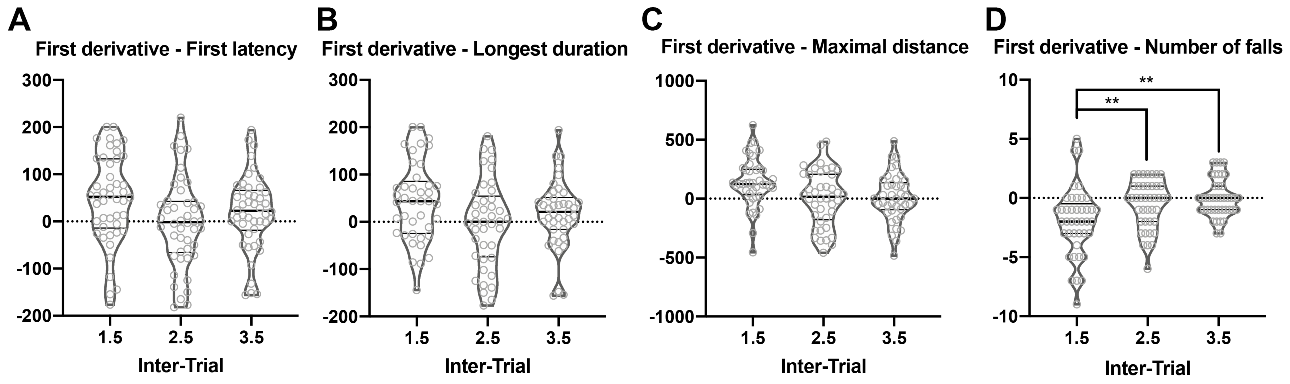

A
First derivative - First latency
300
200
100
0
-100
-200
1.5
2.5
3.5
Inter-Trial
B
First derivative - Longest duration
300
200
100
0
-100
-200
1.5
2.5
3.5
Inter-Trial
C
First derivative - Maximal distance
1000
500
0
-500
-1000
1.5
2.5
3.5
Inter-Trial
D
First derivative - Number of falls
10
5
0
-5
-10
**
**
1.5
2.5
3.5
Inter-Trial
