# Supplementary material for: Four-parameter analysis in modified Rotarod test for detecting minor motor deficits in mice
Source: BMC Biol. 2023 Aug 17;21:177. doi: 10.1186/s12915-023-01679-y (PMC10433596; doi:10.1186/s12915-023-01679-y)
Supplement: Supplementary file 2 — Additional file 2: Table S1. Trials comparison of Sham and TBI mice at different time points. Data at BL, dpo3, dpo14 and dpo21 are analyzed by the Kruskal-Wallis test (non-parametric) and Shapiro-Wilk Test (Normal distribution test): Trial 1 only, Trial 2 only, Trial 3 only, Trial 4 only, Averaged values in Trial 2 to 3 and Averaged Trial 2 to 4. Each Trial had 8 parameters: raw or individual normalized data in First Latency, Longest duration, Maximal distance and Number of falls. Cells filled with grey background in non-parametric test indicate significances, and the darker the grey, the more significant the difference. Asterisks indicate significances: *p < 0.05, **p < 0.01, ***p < 0.001, ****p < 0.0001. Biggest motor deficits within the TBI group showed at dpo14 compared to BL testing detected in all the trials. Starting from Trial 3, we can detect significant difference in 4 parameters not only within the TBI group, but also at the same time point between the TBI and the Sham group: Trial 3 only, Trial 4 only, Averaged Trial 2 to 3 and Averaged Trial 2 to 4. Averaged Trial 2 to 4 showed more sensitive and normal distributed readouts able to detect difference between Sham and TBI at dpo21. With raw data from 4 parameters, data are highly variable in the group (non-normally distributed), we cannot find any difference between TBI and Sham group except a slight difference at BL testing detected by Maximal distance (p = 0.043). [file 12915_2023_1679_MOESM2_ESM.pdf]

		Kruskal-Wallis test followed by post-hoc Dunn's test																Shapiro-Wilk test							
		Sham						TBI						Sham vs. TBI				Sham				TBI			
		BL vs. dpo4	BL vs. dpo14	BL vs. dpo21	dpo4 vs. dpo14	dpo4 vs. dpo21	dpo14 vs. dpo21	BL vs. dpo4	BL vs. dpo14	BL vs. dpo21	dpo4 vs. dpo14	dpo4 vs. dpo21	dpo14 vs. dpo21	BL	dpo4	dpo14	dpo21	BL	dpo4	dpo14	dpo21	BL	dpo4	dpo14	dpo21
**Trial 1**	First Latency - Raw	ns	ns	ns	ns	ns	ns	*	*	ns	ns	ns	ns	ns	ns	ns	ns	ns	ns	ns	ns	*	ns	ns	ns
	Longest duration - Raw	ns	ns	ns	ns	ns	ns	ns	*	ns	ns	ns	ns	ns	ns	ns	ns	ns	ns	ns	ns	*	ns	ns	ns
	Maximal distance - Raw	ns	ns	ns	ns	ns	ns	ns	*	ns	ns	ns	ns	ns	ns	ns	ns	ns	ns	ns	*	ns	ns	*	ns
	Number of falls - Raw	ns	ns	ns	ns	ns	ns	*	*	ns	ns	ns	ns	ns	ns	ns	ns	*	*	ns	ns	ns	*	ns	ns
	First Latency - %	ns	ns	ns	ns	ns	ns	ns	*	ns	ns	ns	ns		ns	ns	ns		ns	ns	ns		ns	**	ns
	Longest duration - %	ns	ns	ns	ns	ns	ns	ns	ns	ns	ns	ns	ns		ns	ns	ns		ns	ns	ns		ns	**	ns
	Maximal distance - %	ns	ns	ns	ns	ns	ns	ns	*	ns	ns	ns	ns		ns	ns	ns		*	ns	ns		ns	*	ns
	Delta number of falls	ns	ns	ns	ns	ns	ns	ns	*	ns	ns	ns	ns		*	*	ns		*	ns	ns		ns	ns	ns
**Trial 2**	First Latency - Raw	ns	ns	ns	ns	ns	ns	ns	**	ns	ns	ns	ns	ns	ns	ns	ns	*	ns	ns	ns	*	ns	ns	ns
	Longest duration - Raw	ns	ns	ns	ns	ns	ns	ns	**	*	ns	ns	ns	ns	ns	ns	ns	ns	*	ns	ns	ns	ns	ns	*
	Maximal distance - Raw	ns	ns	ns	ns	ns	ns	ns	**	ns	ns	ns	ns	ns	ns	ns	ns	ns	ns	ns	ns	*	ns	ns	ns
	Number of falls - Raw	ns	ns	ns	ns	ns	ns	ns	**	ns	ns	ns	ns	ns	ns	ns	ns	ns	ns	ns	ns	****	**	*	**
	First Latency - %	ns	ns	*	ns	ns	ns	*	**	ns	ns	ns	ns		ns	ns	ns		ns	ns	ns		***	***	***
	Longest duration - %	ns	ns	*	ns	ns	ns	*	***	*	ns	ns	ns		ns	ns	ns		ns	ns	ns		***	ns	ns
	Maximal distance - %	ns	ns	*	ns	ns	ns	*	***	ns	ns	ns	ns		ns	ns	ns		***	ns	ns		ns	**	ns
	Delta number of falls	ns	ns	ns	ns	ns	ns	ns	**	ns	ns	ns	ns		ns	ns	ns		ns	ns	ns		ns	ns	****
**Trial 3**	First Latency - Raw	ns	ns	ns	ns	ns	ns	ns	ns	ns	ns	ns	ns	ns	ns	ns	ns	ns	*	ns	ns	**	*	ns	ns
	Longest duration - Raw	ns	ns	ns	ns	ns	ns	ns	ns	ns	ns	ns	ns	ns	ns	ns	ns	ns	ns	ns	ns	*	*	ns	ns
	Maximal distance - Raw	ns	ns	ns	ns	ns	ns	ns	ns	ns	ns	ns	ns	*	ns	ns	ns	ns	ns	ns	ns	ns	*	ns	ns
	Number of falls - Raw	ns	ns	ns	ns	ns	ns	ns	*	ns	ns	ns	ns	ns	ns	ns	ns	ns	ns	*	ns	****	**	***	***
	First Latency - %	ns	ns	ns	ns	ns	ns	ns	ns	ns	ns	ns	ns		ns	*	ns		ns	ns	ns		ns	*	**
	Longest duration - %	ns	ns	ns	ns	ns	ns	ns	ns	ns	ns	ns	ns		ns	*	ns		ns	ns	ns		ns	*	***
	Maximal distance - %	ns	ns	ns	ns	ns	ns	ns	ns	ns	ns	ns	ns		*	*	ns		ns	ns	ns		ns	ns	*
	Delta number of falls	ns	ns	ns	ns	*	*	ns	*	ns	ns	ns	ns		**	**	ns		ns	ns	ns		***	**	*
**Trial 4**	First Latency - Raw	ns	ns	ns	ns	ns	ns	ns	ns	ns	ns	ns	ns	ns	ns	ns	ns	*	ns	ns	ns	**	ns	ns	ns
	Longest duration - Raw	ns	ns	ns	ns	ns	ns	ns	ns	ns	ns	ns	ns	ns	ns	ns	ns	ns	ns	ns	ns	***	ns	ns	ns
	Maximal distance - Raw	ns	ns	ns	ns	ns	ns	ns	*	ns	ns	ns	ns	ns	ns	ns	ns	ns	*	ns	ns	**	ns	*	ns
	Number of falls - Raw	ns	ns	ns	ns	ns	ns	ns	*	ns	ns	ns	ns	ns	ns	ns	ns	**	ns	ns	***	****	***	**	****
	First Latency - %	ns	ns	ns	ns	ns	ns	ns	*	ns	ns	ns	ns		ns	*	ns		ns	ns	ns		ns	****	****
	Longest duration - %	ns	ns	ns	ns	ns	ns	ns	**	ns	ns	ns	ns		ns	*	ns		ns	*	ns		ns	***	ns
	Maximal distance - %	ns	ns	ns	ns	ns	ns	ns	**	ns	*	ns	ns		ns	ns	ns		ns	**	ns		ns	****	ns
	Delta number of falls	ns	ns	ns	ns	ns	ns	ns	**	ns	ns	ns	*		**	*	ns		ns	ns	**		***	***	****
**Averaged Trial 2 and 3**	First Latency - Raw	ns	ns	ns	ns	ns	ns	ns	**	ns	ns	ns	ns	ns	ns	ns	ns	ns	ns	ns	ns	ns	ns	ns	ns
	Longest duration - Raw	ns	ns	ns	ns	ns	ns	ns	*	ns	ns	ns	ns	ns	ns	ns	ns	ns	ns	ns	ns	ns	ns	ns	ns
	Maximal distance - Raw	ns	ns	ns	ns	ns	ns	ns	**	ns	ns	ns	ns	ns	ns	ns	ns	ns	ns	ns	ns	ns	ns	ns	ns
	Number of falls - Raw	ns	ns	ns	ns	ns	ns	ns	**	ns	ns	ns	ns	ns	ns	ns	ns	ns	ns	ns	ns	****	**	*	***
	First Latency - %	ns	ns	ns	ns	ns	ns	ns	***	*	*	ns	ns		ns	ns	ns		**	ns	ns		*	****	****
	Longest duration - %	ns	ns	ns	ns	ns	ns	ns	**	*	ns	ns	ns		ns	*	ns		ns	ns	ns		ns	ns	ns
	Maximal distance - %	ns	ns	ns	ns	ns	ns	ns	***	*	ns	ns	ns		ns	**	ns		*	ns	ns		ns	ns	ns
	Delta number of falls	ns	ns	ns	ns	ns	ns	ns	**	ns	ns	ns	ns		ns	*	ns		ns	ns	ns		*	*	****
**Averaged Trial 2, 3 and 4**	First Latency - Raw	ns	ns	ns	ns	ns	ns	ns	**	ns	ns	ns	ns	ns	ns	ns	ns	ns	ns	ns	ns	ns	ns	ns	ns
	Longest duration - Raw	ns	ns	ns	ns	ns	ns	ns	*	ns	ns	ns	ns	ns	ns	ns	ns	ns	*	ns	ns	ns	ns	ns	ns
	Maximal distance - Raw	ns	ns	ns	ns	ns	ns	ns	**	ns	ns	ns	ns	ns	ns	ns	ns	ns	ns	ns	ns	ns	ns	ns	ns
	Number of falls - Raw	ns	ns	ns	ns	ns	ns	ns	**	ns	ns	ns	ns	ns	ns	ns	ns	ns	ns	ns	ns	****	**	**	***
	First Latency - %	ns	ns	ns	ns	ns	ns	ns	***	*	ns	ns	ns		ns	*	ns		ns	ns	ns		ns	ns	ns
	Longest duration - %	ns	ns	ns	ns	ns	ns	ns	***	*	*	ns	ns		ns	**	ns		ns	ns	ns		ns	ns	ns
	Maximal distance - %	ns	ns	ns	ns	ns	ns	ns	**	*	**	ns	ns		ns	**	*		ns	ns	ns		ns	ns	ns
	Delta number of falls	ns	ns	ns	ns	ns	ns	ns	***	ns	*	ns	ns		ns	**	ns		ns	ns	ns		**	**	****
