# Supplementary material for: Four-parameter analysis in modified Rotarod test for detecting minor motor deficits in mice
Source: BMC Biol. 2023 Aug 17;21:177. doi: 10.1186/s12915-023-01679-y (PMC10433596; doi:10.1186/s12915-023-01679-y)
Supplement: Supplementary file 3 — Additional file 3: Fig. S2. The difference between real-time counting and counting from recorded video. A The difference in the falling time by 2 different methods (Difference = falling time counted real-time - falling time counted from recorded video) from in total of 6 5-min videos (n = 6 animals). B The time difference between the time to pick the mice and the time of falling (Pick-Fall), the time of releasing the mice from hands to the time of falling (Release-Fall) and the time difference between Release and pick (Release-Pick) in a total of 30 periods. The black lines stretched from violin plots were defined as third quartile, median and first quartile. [file 12915_2023_1679_MOESM3_ESM.pdf]

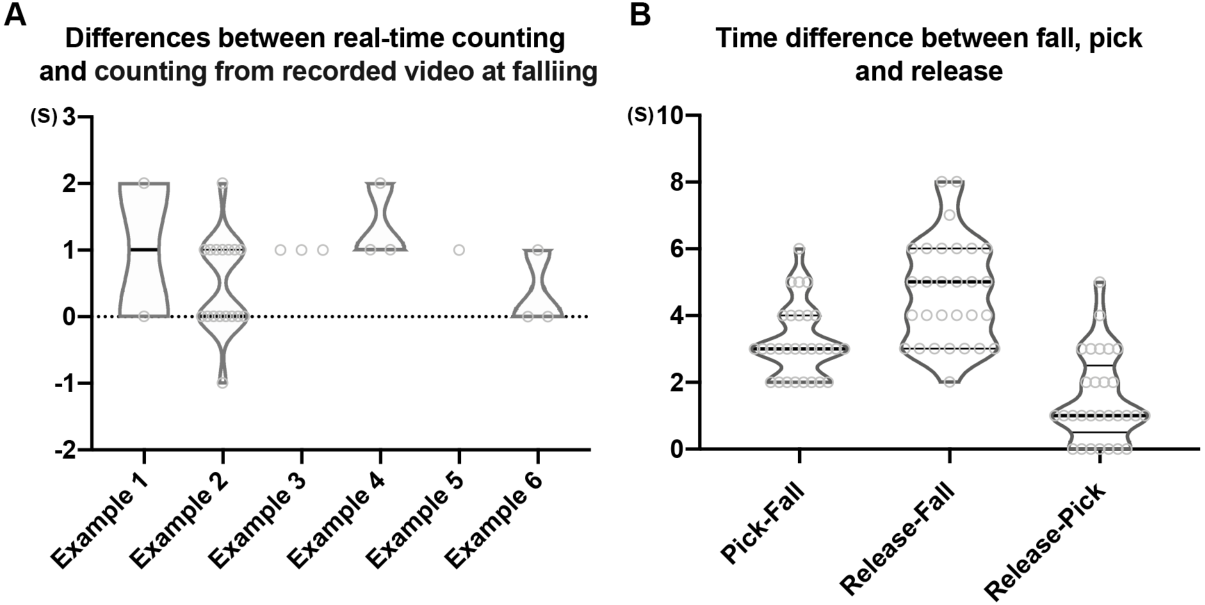
A
Differences between real-time counting and counting from recorded video at falliing
(S)
3
2
1
0
-1
-2
Example 1
Example 2
Example 3
Example 4
Example 5
Example 6
B
Time difference between fall, pick and release
(S)
10
8
6
4
2
0
Pick-Fall
Release-Fall
Release-Pick
